# Buffering or not working: group counseling for depression and loneliness among boarding primary school students

**DOI:** 10.3389/fpubh.2024.1462634

**Published:** 2025-01-03

**Authors:** Peng Wang, Junchi Ma, Longlong Du, Qiulian Xing, Xinyu Cheng, Mingzhu Zhang, Fei Geng, Yuanxin Zheng, Fangxiao Zheng, Mei Tian

**Affiliations:** ^1^School of Education, Wenzhou University, Wenzhou, China; ^2^College of Psychology, Shandong Normal University, Jinan, China; ^3^Normal College, Weifang Institute of Technology, Weifang, China; ^4^School of Digital Creativity, Shandong College Of Electronic Technology, Jinan, China

**Keywords:** boarding primary school students, group psychological counseling, depression, loneliness, intervention

## Abstract

**Introduction:**

Due to the acceleration of modern life rhythm, students with developing minds are susceptible to negative external influences, leading to a growing concern for their mental health. Boarding primary school students have limited interaction with relatives compared to their non-boarding counterparts, rendering them more prone to feelings of depression and loneliness, resulting in various negative emotions. Therefore, our study aimed to explore the effects of group counseling interventions on reducing depression and loneliness among adolescents.

**Methods:**

The study analyzed loneliness and depression before and after the intervention in eight randomly selected classes of fifth-grade students at a boarding school in a region of Shandong Province, which were divided into an experimental group and a control group.

**Results:**

Within the experimental group, there were no substantial variations in loneliness and depression levels. In the control group, post-test depression results significantly surpassed pre-test scores (*p* = 0.046), though loneliness levels did not differ significantly. Conversely, the experimental group displayed significantly reduced post-test depression levels compared to the control group (*p* = 0.037), with no significant variance in loneliness.

**Conclusion:**

The findings indicated that group psychological counseling mitigates depression in the experimental group to a certain extent, affirming the efficacy of the intervention. The study demonstrated that group counseling alleviates depression in boarding students, emphasizing the value of the intervention.

## 1 Introduction

As primary school students’ mental health education receives increasing attention, many studies on self-perception, emotional management, and social interactions have emerged. A primary task of mental health education is to train students to have a healthy and positive mental state that can help them successfully deal with the difficulties and frustrations they encounter in school and family. Although students’ physical health can be monitored through regular school physical examinations, their mental health problems are not easily detectable. A boarding school is an educational institution where the majority or all of the students reside on campus during the academic year. The term “boarding” refers to the provision of accommodation and meals, often referred to as “bed and board” (1). Boarding schools are typically chosen by families with absent parents or students who live far away from home, as it eliminates the need for a daily commute. The primary school phase is crucial for children’s cognitive development, especially in their transition from concrete to abstract thinking. Additionally, this period is important for the development of self-identity, recognition of individual differences, and the gradual formation of a comprehensive self-evaluation framework. In this context, students learn to build interpersonal relationships and integrate into social groups. During this critical stage, children acquire essential skills for successful integration into larger societal frameworks, but most parents of boarding primary school students cannot often accompany those students inside the school, and some such students live with distant relatives; thus, it is difficult for them to obtain support and help from parents and family (2). Research has shown that the boarding system positively influences the development of self-esteem, moral norms, self-concept, socialization, peer relationships, self-evaluation, teacher-student relationships, and life-planning abilities of primary school students. However, it negatively impacts their understanding of social roles, limits their social circles, and hinders their mental health development (3–5). In addition, due to the particularity of the boarding school environment, such as in cases of long-term closure of the school environment, primary students can easily experience loneliness, depression and other negative psychological problems over time. Boarding school for students is a “double-edged sword”; on the one hand, for students who cannot receive family care, boarding school can help them obtain better care and attention than their families (6), while on the other hand, boarding school does not pay attention to students’ emotional problems, peer relationships and loneliness (7), and some studies have noted that boarding school students experience higher anxiety, stress and loneliness than non-boarding school students (8).

Group psychological counseling is a way to guide homogenous group members to promote and influence each other. Some scholars have used group psychological counseling to intervene in cases of depression, loneliness and anxiety among different groups, and the results have proven the feasibility of group counseling interventions (9). Simultaneously, some scholars have also demonstrated the applicability of group counseling interventions for primary school students, such as by improving their loneliness, depression, peer relationships and social anxiety (10, 11). In previous studies, scholars have paid more attention to depression and loneliness, but few group counseling studies have focused on depression and loneliness among boarding primary school students. Therefore, this paper takes boarding primary school students as its research object, focusing on their negative emotions such as depression and loneliness, and uses group psychological counseling based on the theory of positive psychology, the Satya model and non-violent communication to intervene with the aim of effectively improving their mental health state.

### 1.1 Group psychological counseling

#### 1.1.1 Overview of group psychological counseling

Group psychological counseling originated in the West. At present, scholars hold different opinions regarding the definition of group counseling, and no unified conclusion has yet been reached. Chaplin (12) claimed that group counseling includes various forms of group therapy. In the process of therapy, the therapist acts as a leader and the patient as a member, and both parties can achieve catharsis and therapeutic effects by listening to other people’s problems and solving them. Gazda et al. (13) proposed that group counseling is a dynamic interpersonal process. Group members can improve their understanding and acceptance of values and goals through group interaction and can learn and change certain attitudes and behaviors.

In recent years, major schools have also begun to combine group counseling with their own theories, such as group counseling based on focus solutions (14), group counseling based on Bandura’s self-efficacy theory (15), group counseling according to positive psychology and group counseling based on the Satir model (16). The group psychological counseling activities on which this paper focuses are based mainly on positive psychology, the Satir model and non-violent communication, and the group counseling activities are further designed for boarding primary school students. These factors are described successively in the following sections.

#### 1.1.2 Group counseling according to positive psychology

Positive psychology is a new field of psychological research that emerged in the United States in the 1990s. The definition of positive psychology is evolving. Positive psychology is the study of the conditions and processes that contribute to the flourishing or optimal functioning of people, groups, and institutions (17). Under the influence of the positive psychology concept, a form of group counseling based on positive psychology gradually emerged and developed in the practice of group counseling. Some scholars have used the theory of positive psychology to implement group counseling. The results showed that this method can not only effectively alleviate the individual’s depressive symptoms and reduce the individual’s levels of loneliness and inferiority but also improve the individual’s life satisfaction. Simultaneously, it also has a significant effect on improving the individual’s self-esteem level and the mental health level of the mothers of children with intellectual disability (18–20). In addition, some scholars have implemented positive psychological counseling on the basis of theory. For example, Steve et al. (21) used positive psychological group counseling based on Adlerism to intervene in the youth group living in the treatment center. The results showed that the optimism and happiness of this group were significantly improved. Based on this research, it can be observed that positive psychological group counseling is an effective way to intervene in individuals’ mental health and improve their positive quality. Therefore, this paper takes this approach as one of its theoretical foundations for designing a group counseling course to address boarding primary school students’ depression and loneliness.

#### 1.1.3 Group counseling according to the Satir model

The Satir model developed by Virginia Satir is aimed mainly at family therapy. Satir model is growth-oriented and people-oriented, emphasizes caring for people, and comprehensively uses a variety of psychotherapy techniques with different orientations. Because of its positive ideas and high level of practicality, various countries have gradually introduced the Satir model into practice (22).

Psychological counseling according to the Satir model has achieved significant practical results worldwide. Some scholars have proven that the Satir model can effectively improve the state of depressed patients (23), and in the context of the COVID-19 epidemic, group intervention based on the Satir model can effectively alleviate the anxiety and depression of adolescents and improve their life satisfaction and resilience (23). Many Chinese scholars have combined the Satir model with group psychological counseling. Studies have proven that Satir group counseling can improve the independent development ability and interpersonal relationships of children with learning difficulties (24, 25) as well as their mental health level. Research has also confirmed that the Satir model of group counseling is suitable for primary school students (26).

Lin (26) summarized that the intervention techniques used in group counseling in the Satir model, which at present mainly include meditation, family remodeling, personalized dance and interactive component intervention techniques. Meditation can help group members quickly detach from high-pressure states of mind, adjust their mood, calm their internal dialog, focus their energy, and open up the right hemisphere of the brain for emotion and intuition. This process puts individuals in touch with themselves, integrating the resources of all parts of their being, which can catalyze a state of entrancing and promote internal change through positive guidance and suggestion (27). Individualized dances typically end with participants dancing on stage, minimizing the exposure of another side of themselves (28). In the design of group activities in this paper, meditation techniques and personalized dance are used to reduce the depression and loneliness of primary school students and to help them to re-recognize themselves and discover their uniqueness and advantages.

#### 1.1.4 Group counseling according to non-violent communication

Non-violent communication refers to a dialog between two or more parties that is based on mutual respect and trust, expressing oneself bravely and truly, trying to listen to others while being responsible for one’s own behavior and performance, and aiming to establish harmonious interpersonal relationships (29). Non-violent communication can effectively address people’s emotional and behavioral problems, promote interpersonal harmony and help individuals adapt to the external environment more effectively. The research subject of this paper is boarding primary school students, who are young and lack communication skills. Due to the particularity of their living and learning environment, boarding school students spend less time with their families, and most of their time is occupied by peer communication and teacher-student interaction. If they do not master good communication skills, they tend to experience more serious depression and loneliness (30). Based on previous studies, non-violent communication is an effective way to address emotional and behavioral problems (31, 32). Therefore, this study designed this group counseling activity based on this theory to address the depression and loneliness of boarding primary school students.

### 1.2 Related research on depression

The World Health Organization defines depressive disorder, also known as depression, as a common mental disorder characterized by prolonged periods of low mood, or loss of pleasure and interest in activities. Angold and Rutter (33) described depression as a persistent low or bad mood, which is a kind of unhappiness, sadness and mental pain. It is a negative response to adverse events. Sun (34) described depression is a state of mind disorder in which an individual develops persistent low mood, demoralization, diminished interest, lack of energy, anhedonia, and even suicidal thoughts or behaviors, among other clinical features. Compared to depression, depressed mood in almost everyone, and its causes are many and varied, ranging from childhood circumstances to the pressures of life.

In recent years, the ages with depression have gradually decreased, and the incidence of depression among adolescent groups has increased significantly. Depression increases the physical and psychological burdens people face. Studies have shown that parents with depressive symptoms increase the incidence of depression in children. Furthermore, depression can increase the risk of tuberculosis and cardiovascular diseases, and it can lead to sleep disorders or even criminal behavior (35). In addition, depression impacts students’ physical and mental health, thereby affecting their learning and social interaction and significantly increasing their risk of depression in adulthood (36, 37). Some studies have also found that mood problems in middle school students not only affect their current academic performance and interpersonal relationships but also increase their risk of developing major depression and generalized anxiety disorder in adulthood (3). Additionally, several studies have found that children who experience depression early in life are more likely to face emotional problems, such as anxiety, low self-esteem, and negative self-perception, in adulthood (38). Based on this research, depression has many adverse effects on individuals, and primary school students are in a critical period of development, thereby requiring our special attention.

#### 1.2.1 Research on depression among boarding primary students

Primary school students are undergoing a period of rapid physical and psychological development. Due to societal development and changes in educational methods, all of these factors place a certain degree of psychological pressure on primary school students, and it is easy for them to encounter various mental health problems, among which depression is one of the more common. Many scholars have successively studied depression among primary school students. For example, Ford et al. (39) discussed the relationship between primary school students’ depression and school level. The results showed that the economic development of the school area was positively correlated with students’ depression. Zhang et al. (40) studied the current situation of primary school students’ depression in Chengdu and found that excessive study pressure, family and parenting style were all risk factors for depression.

However, boarding school students’ lives involve great changes and a lack of contact with their parents and families, which are not conducive to the development of their values, socialization and mental health. For example, some scholars have found that the mental health of boarding school students is worse than that of non-boarding school students (41). An Australian study found that boarding school students experienced more anxiety and perceived stress than non-boarding school students (8). Accordingly, many studies have focused on boarding school students, but relatively few studies have focused on boarding primary students. Pupils are undergoing a period of rapid physical and mental development and thereby require more attention. Therefore, this paper chooses boarding primary students as its research subject and examines further interventions aimed at them.

#### 1.2.2 Research on group psychological counseling for depression among boarding primary students

In previous research on group counseling and interventions for depression, scholars have focused more closely on adult groups. For example, Ekrami et al. (42) conducted a group counseling intervention on pregnant women. As a result, the depression of the experimental group was significantly lower than that of the control group. Some scholars have also intervened in the depression of college students. For example, some scholars have used psychological counseling and group counseling to intervene in the depression of college students, and the results showed that the anxiety and depression of those students were reduced after the intervention (43). Furthermore, a few scholars have intervened in the depression of primary school students. In summary, few studies have investigated depression interventions for primary school students, especially boarding primary school students, and group counseling interventions in this context have been even fewer. However, studies on depression intervention through group counseling all showed significant results.

### 1.3 Related research on loneliness

The word “loneliness” is drawn from clinical medicine. At first, this notion referred to a kind of psychological dysfunction. Subsequently, scholars introduced loneliness into the research category of “social psychology.” Weiss (41) claimed that loneliness was a subjective experience and a psychological gap in daily communication activities. Such a subjective experience is unpleasant and painful, and it involves a psychological feeling that is not accepted by the members of the group or others. Simultaneously, it also indicates that the interpersonal and intimate relationship network of the individual in question has collapsed.

#### 1.3.1 Research on the loneliness of boarding primary school students

In recent years, due to the intensification of social competition and changes in lifestyle and parental rearing patterns, modern children experience loneliness to a greater or lesser degree. Children’ loneliness refers to the bad mood generated by pupils’ interpersonal communication, which leads to many negative results, such that these pupils do not experience a sense of belonging to the team. Over time, they lose their self-confidence and self-esteem, leading to serious social barriers and affecting their normal study and life (44). Loneliness in childhood can shape an individual’s personality traits. Children who are chronically lonely may become introverted, sensitive, and withdrawn, and may struggle with socializing. These personality traits may carry over into adulthood, affecting their social skills and relationships (45, 46).

In recent years, some scholars have studied the prediction indicators and influencing factors of primary school students’ loneliness, in the context of the relationship between loneliness and peer relationships. The results of these studies showed that the value affirmation of same-sex friends and peer acceptance level are negatively correlated with children’s loneliness. The teacher-student relationship is also related to pupils’ loneliness. The more harmonious the teacher-student relationship is, the lower the pupils’ loneliness (47). Simultaneously, internal subjective factors such as personality traits and the attachment style of primary school students can also affect loneliness (48). Qu (49) found that levels of emotion and openness can significantly and positively predict individual loneliness. Loneliness is also related to family function and social support. In summary, based on the above research, we can conclude that peer relationships, teacher-student relationships and personal traits are related to the loneliness of primary school students, which can encourage us to pay special attention to these factors in the process of developing an intervention for loneliness in this study.

#### 1.3.2 Research on group psychological counseling for loneliness among boarding primary students

In extensive practical research that has been conducted in recent years, group counseling methods have been found to play an important role in improving students’ psychological and behavioral problems (50). To alleviate children’s loneliness more effectively, many scholars have designed various psychological counseling activities, all of which have achieved good results by helping children better integrate into groups, be accepted by group members and experience a sense of belonging. For example, cognitive behavior group counseling has a significant effect on reducing children’s level of time anxiety (51). Yin (52) used group sand play therapy and painting group counseling to intervene in the loneliness of primary school students. By analyzing students’ sand play pictures and classic “house-tree-person” paintings, the results proved that these interventions can effectively reduce the loneliness of primary school students. Similarly, to reduce the loneliness of primary school students, Zhang and Yue (53) designed a series of group activities supported by the theory of positive psychology. The results proved that these activities had a good effect on improving the social anxiety, social phobia and avoidance of students in grades 5 and 6. Furthermore, studies have investigated group counseling interventions for groups with bad peer relationships and groups with interpersonal difficulties in boarding schools with the aim of improving the subjects’ interpersonal problems, increasing their level of self-esteem and ultimately reducing their loneliness. In summary, group counseling interventions for the loneliness of primary school students have achieved good results. Group counseling has a positive effect on improving group common psychological problems. With regard to the particularity of the study subject, boarding primary school students face more parent–child relationships and interpersonal communication pressure than ordinary primary school students (54, 55). Therefore, this paper also uses the same form of intervention with the aim of reducing the loneliness of boarding primary school students.

### 1.4 Statement of the problems

The characteristics of boarding primary school students who are separated from their families and the trend toward a younger age of depression have caused this group to receive increasing amounts of attention; in terms of loneliness, social adaptation, emotional state, interpersonal communication and other aspects, the situation of such students is not optimistic, and some students have a strong tendency toward depression and anxiety. At present, intervention methods based on group psychological counseling for various psychological problems are in a stage of rapid development. On the basis of social learning theory and collective dynamics theory, many scholars have successively integrated positive psychology, developmental psychology and other theories to achieve good results (53); however, according to the extant literature, fewer psychological interventions were used for boarding primary school students in the past, and fewer group counseling studies have focused on depression and loneliness among boarding primary school students. Therefore, on the basis of previous theories, this paper proposes combining positive psychology, the Satya model, non-violent communication and group counseling to design a set of group psychological counseling programs to intervene in the depression and loneliness of boarding primary school students with the aim of helping them develop good psychological quality so that they can cope with new tasks in learning, life and the environment more effectively. To a certain extent, this study enriches and develops the theoretical basis of group counseling, provides new theoretical support for the development of group counseling in the future, and offers a practical reference for the development of psychological counseling courses in boarding primary schools. Simultaneously, it also provides an empirical basis for improving the loneliness and depression of boarding primary students, which has important practical significance. Based on the extant research results and relevant theories, this paper proposes the following hypotheses:

1. The levels of depression and loneliness in the pre-test are no significantly differences between the experimental group and the control group.
2. The levels of depression and loneliness of the experimental group in the post-test are significantly lower than those of the control group.
3. In both the pre-test and post-test, there are no significant differences in loneliness and depression symptoms in the control group, while the experimental group’s levels significantly decrease.

## 2 Method

### 2.1 Subjects

The present study selected fifth-grade students from boarding schools in a specific region of Shandong Province. Prior to the commencement of the research, sample size calculation was conducted using G*Power 3.1. A two-tailed significance level of 0.05 was set, and to ensure a statistical power (Power) of at least 0.8, 52 participants were required. Four classes were randomly chosen from the fifth-grade level to serve as the experimental group, and another four classes were designated as the control group. Both the control group and the experimental group were in the same school and took their final exams simultaneously during the post-test period. After eliminating invalid responses, a total of 288 questionnaires were retained. Following reverse scoring for certain items and subsequent replacement of missing values using mean imputation, the data analysis was performed. Among them, there are 158 boys, accounting for 54.9%, and 130 girls, accounting for 45.1%.Ultimately, the number of participants in the experimental group who received group counseling intervention was 144, while the control group, also comprised 144 participants.

### 2.2 Measures

Depression and Loneliness scales were administered as assessment questionnaires to investigate the levels of loneliness and depression among fifth-grade students in boarding schools within the specified region. The specific details of each scale are as follows:

#### 2.2.1 Depression scale

This scale includes 7 questions, which are scored on a 4-point scale. The higher the score is, the higher the level of depression. The Cronbach’s *α* coefficient of this scale is 0.884, and the correlation between this dimension and the total score is 0.374 ~ 0.932. The detailed questionnaire can be found in Appendix 2.

#### 2.2.2 Loneliness scale

This scale includes 24 questions, of which 16 questions are scored on a 5-point scale, while the remaining 8 questions are inserted questions without scoring. The higher the total score is, the higher the level of loneliness. The Cronbach’s *α* coefficient of 16 lonely items is 0.90. The detailed questionnaire can be found in Appendix 3.

### 2.3 Research process

#### 2.3.1 Pre-test of experiment

After discussions with schools, parents, and third-party educational institutions, it was ultimately decided to select fifth-grade students from boarding schools in a specific region as the subjects for this research. Initially, students from eight classes were randomly selected for a questionnaire survey. The questionnaires primarily included the *Depression* and *Loneliness scales*. Based on the questionnaire results, a final decision was made to designate four classes as the participants for the group psychological counseling in this study.

#### 2.3.2 Implement group psychological counseling

The group counseling program spans one semester. The experimental group participated in counseling sessions held on Thursdays and Sundays at 6:40 PM in a designated classroom within a specific school. Each session lasted approximately 90 min. Group counseling activities were implemented for participants in the experimental group. These group counseling activities are structured into 11 lessons. Prior to the commencement of the course, the scenario was introduced, and groups were organized. At the conclusion of the course, each group elected a representative to share their insights, followed by a summary provided by instructor. The group counseling program is primarily grounded in the principles outlined in “The 7 Habits of Highly Effective People” and is designed using the Satir model, non-violent communication, and positive psychology as theoretical foundations. The primary objective of group counseling is to foster positive habits among children.

Lessons 1–4 mainly cultivate children’s active habits, namely, focusing on taking positive action and being responsible for your past, present, and future actions. The activities encourage students not to blame others, to sublate the passive victim role, to create change from the inside out, and to face everything actively. Creating and cherishing one’s life is the most basic decision made by everyone; Lesson 5 aims at cultivating children’s habit of beginning with the ending in mind, namely, the notion that everything has to be created twice, the first in the mind and the second in essence. We must know where we are now and where our destination is. In this way, we can firmly proceed with our mission and declaration and create a culture. Lesson 6 aims at cultivating children’s habit of putting things first, namely, based on the notion that the first important thing is the goal, vision, and the order in which the important things are handled. Secondary matters cannot be put first. No matter how urgent a situation is, we must first put the important matters first. Lesson 7 focuses on cultivating children’s habit of win–win thinking. Win–win refers to a kind of thinking based on reciprocity, mutual respect, and a lack of hostile competition. It encourages us to find a mutually beneficial solution, which is a message, a power. Lesson 8 aims at cultivating children’s habit of knowing each other and understanding themselves; only through effective listening can we facilitate real communication and enhance intimacy. It takes patience and courage to show your heart to others. Lessons 9–10 focus on cultivating children’s habit of integrative efficacy, unity and efficiency with the aim of achieving 1 + 1 > 2 effectiveness, which involves a team and a relationship that implements unity, abandons adversarial attitudes and does not compromise. Instead, they seek creative cooperation. Lesson 11 focuses on cultivating children’s ability to engage in constant renewal, which refers to constantly updating oneself physically, mentally and emotionally with each passing day with the aim of avoiding aging and fatigue, leading oneself to a new journey and starting a new struggle. The group counseling designed in this study primarily aims to address the problems of depression and loneliness. It is conducted by professional psychological institutions and counselors, with the schedule and class content tailored to specific situations. The content of the group counseling sessions is diversified and presented in various forms. The detailed program can be found in Appendix 1.

#### 2.3.3 Post-test of experiment

After 11 group psychological counseling activities, the experimental group and the control group completed the Depression and Loneliness Scale once again.

#### 2.3.4 Data analysis methods

SPSS 22.0 software was used for quantitative analysis of the results regarding the experimental group and control group.

If properly applied, repeated measurements can be simplified at the design stage to only two time points, making the process more efficient and cost-effective. Regarding analysis, the t-test is the simplest choice as it compares differences in pre- and post-test scores or scores obtained by group (i.e., experimental, control). In this study, we administered the test twice, before and after, to the students in the residential school, and the data were analyzed using a paired samples *t*-test. However, pre-test equivalence (no between-group differences in pre-test scores) is a necessary assumption when analyzing differences in post-test scores using *t*-tests. Although pre-test equivalence can be assumed in the case of a randomized design, pre-test differences between groups should be examined before performing a *t*-test on post-test scores. Therefore, we also performed a pre-test equivalence test, the procedure for which is described in section 3.1.

## 3 Results

### 3.1 Difference test of pre-test average of the experimental group and control group

The average and standard deviation of the test results of the experimental group and the control group before group counseling are shown in Tables 1, 2. After eliminating extreme values, an independent sample *T*-test was performed on the pre-test results of the experimental group and the control group. The results showed that there were no significant differences in all dimensions, thus indicating that the levels of all dimensions of the two groups of subjects were parallel during the pre-test.

**Table 1:** *T*-test results of pre- and post-test depression in the experimental and control groups.

Pre-test vs. Post-test	*t*	*p*
Experimental group	−0.282	0.778
Control group	−2.016	0.046*

Experimental group vs. Control group	*t*	*p*
Pre-test	−1.163	0.246
Post-test	−2.093	0.037*

*p < 0.05, **p < 0.01, ***p < 0.001.

**Table 2:** *T*-test results of pre- and post-test loneliness in the experimental and control groups.

Pre-test vs. Post-test	*t*	*p*
Experimental group	0.503	0.616
Control group	−0.570	0.570

Experimental group vs. Control group	*t*	*p*
Pre-test	−1.408	0.160
Post-test	−1.744	0.082

### 3.2 Difference test of post-test average of experimental group and control group

After eliminating extreme values, the post-test of the experimental group and the control group was tested using an independent sample *t*-test. The results showed that there was a significant difference in the depression dimension (*p* = 0.037), indicating that the level of depression exhibited by the post-test experimental group was significantly lower than that of the control group; furthermore, there were no difference in the scores on the loneliness scale, indicating that there were no significant differences in the level of loneliness between the experimental and control groups at post-test. See Tables 1, 2 for details.

### 3.3 Difference test of average pre- and post-test scores in the control group

See Tables 1, 2 for the average and standard deviation of the pre- and post-test results of the control group without the group counseling intervention. After eliminating extreme values, the pre- and post-test results of the control group were tested using *t*-tests. The results showed that there was a significant difference in the depression dimension between the control group (*p* = 0.046), thus indicating that compared with the pre-test, the post-test depression level of the control group was significantly improved and that there was no significant difference in the children’s loneliness scale.

### 3.4 Difference test of the average pre- and post-test in the experimental group

See Tables 1, 2 for the average and standard deviation of the test results before and after group counseling in the experimental group. After eliminating extreme values, the pre- and post-test results of the experimental group were tested using *t*-tests. The results showed that there were no significant differences between the depression scale and the loneliness scale.

### 3.5 Trends in the levels of depression and loneliness in the experimental and control groups before and after the group counseling intervention

In conclusion, according to the results shown above, the group counseling activities designed in this study aimed to improve the depression and loneliness of boarding primary students, but the results showed that these group counseling activities only had a buffering effect on the depression of boarding primary students and did not worsen subjects’ depression; however, they had no significant effect on subjects’ loneliness.

## 4 Discussion

### 4.1 Analysis of group counseling intervention results for boarding primary students

A review of the existing literature reveals that current research on the mental health of elementary school students does not differentiate between various mental health problems, such as depression, loneliness, and anxiety.

According to the results of this study, the hypotheses were tested as follows: Hypothesis one showed no significant difference between the groups in the pre-test. Hypothesis two was partially supported, with a significant increase in the level of depression but no difference in the feeling of loneliness in the control group at the post-test. Hypothesis three was partially supported, showing a significant increase in the feeling of depression over time. Hypothesis four was partially supported, showing a significant decrease in the level of depression but no difference in the feeling of loneliness in the experimental group’s post-test compared to the control group. After the group counseling intervention, there were no significant differences in depression and loneliness in the experimental group compared to the pre-test, whereas the control group exhibited increased levels of depression and no differences in loneliness from the pre-test. These results indicate that, over time, boarding elementary school students’ feelings of depression can rise significantly as final exams approach, which requires teachers’ attention. The group counseling intervention had a buffering effect on the experimental group’s depression, preventing it from worsening but not significantly alleviating the already present symptoms. This finding suggests that the group counseling program designed in this study can improve the depression of boarding elementary school students, thus differing from previous research results concerning group counseling interventions. For example, scholars have used group counseling to intervene in the depression of older adults women and pregnant women, and the results all showed that this intervention can effectively improve the depressive symptoms of the group (42). Other scholars have used group counseling to intervene in the poor peer relationships of boarding primary school students, and the results of research on that topic have also shown that this intervention can reduce their loneliness (7).

### 4.2 Discussion of the reasons why group counseling can buffer depression but not loneliness

Comprehensive group psychological counseling can buffer the depression of boarding primary school students but not their loneliness. Possible reasons for this difference are as follows.

Firstly, concerning the timing of the questionnaire administration, during the post-test phase, this group was in the midst of final exams, a period that naturally brings about stress for students. Those who had undergone group counseling had acquired strategies to cope with stress and improve negative emotions throughout the process. As a result, this could mitigate the negative emotional impact caused by external stressful events, particularly in terms of depression and loneliness. On the other hand, the control group students, not having participated in group counseling, were comparatively less equipped in this regard, rendering them more susceptible to negative influences.

Second, in terms of the design of group-assisted programs, this paper analyzed previous group-assisted programs for depression and loneliness and found that in group-assisted intervention programs for depression, scholars have given more attention to cognitive and emotional aspects. For example, Zhu (44) emphasized the importance of emotional experience. In this paper, the transformation of negative cognition and the understanding of emotions are also involved in the design of the auxiliary plan of the group. Therefore, it can be speculated that the group’s depression is alleviated for this reason. In group-assisted intervention programs for loneliness, scholars have all focused on interpersonal communication, advocating the cultivation of group social skills and the enhancement of social ability with the aim of promoting a virtuous cycle of interpersonal communication. For example, Guo (11) and others all focused on interpersonal communication when designing group-assisted intervention programs. Examples include “Squares and Circles of Communication,” “The Art of Interpersonal Communication” and “The ABC’s of Communication.” With regard to the scheme used in this paper, although it involves teamwork, win–win thinking and other aspects, it rarely involves interpersonal activities. Therefore, it can be speculated that the imperfection of the team-assisted scheme may also lead to the lack of a significant difference in loneliness.

### 4.3 Limitations and directions for future research

#### 4.3.1 Limitations

The limitations of this paper are as follows: During the post-test of this study, the subject group faced the prospect of final exam, which may have had an impact on our research. A self-report scale was used to collect data, and some primary school students did not cooperate with the research seriously but rather merely completed the scale in a perfunctory manner, which affected the quality of follow-up data analysis. We selected fifth-grade students from boarding schools in a specific region. This choice of subjects is limited and thus cannot represent the situation of pupils in this school to a certain extent, nor can it explain the overall situation of boarding primary school students in a contemporary social background. Due to the limitations associated with protecting the information of the research subjects and the conditions of the hardware used, no complete recording of the group counseling activities is available. If these activities could be recorded, future in-depth analyses could be more convincing. Due to the large number of subjects involved, our group counseling activities were divided into two simultaneous classes. Although the content of group counseling activities in each class was the same, the teachers in the two classes were different, and their leadership styles were slightly different, which also led to some differences in the effect of the activities on children.

#### 4.3.2 Directions for future research

This study introduces a novel theoretical framework by integrating positive psychology, the Satir model, and non-violent communication into group counseling to address depression and loneliness issues among boarding primary school students. Furthermore, the study’s findings offer reference points for those engaged in practical ‘buffering’ effects of group counseling, with potential for further exploration of the theoretical mechanisms that underlie such effects. It also demonstrates the effectiveness of conducting group counseling activities for boarding primary school students, showcasing practicality. This approach can be extended to the entire school or other regions in the future, integrating group counseling techniques into regular psychological education classes to maximize their impact and enhance the psychological well-being of elementary school students.

### 4.4 Suggestions

In summary, to improve the mental health of boarding primary school students, we must pool all forces. Schools can improve in many ways, not only by providing good “hard conditions equipment,” including beautiful campus environments, delicious meals, and comfortable accommodation environments but also by improving the mental health education teams in schools and the ways in which students can ask for help, such as through a “soul mailbox.” The soul mailbox is an innovative tool that combines counseling and writing therapy. It provides an open, anonymous, and safe space for students to express their inner feelings and worries, pour out their emotions, and receive support and guidance from professionals. Students should play their main role well, learn some self-help methods to address psychological problems, and improve their quality and ability in all respects.

## 5 Conclusion

Based on this research, we can conclude that the depression of boarding primary school students gradually increases over time, and through group psychological counseling intervention, their depressive symptoms can be alleviated; thus, their mental health level can be promoted. Therefore, this method has a certain degree of practical significance and is worthy of further popularization and application.

## Data Availability

The original contributions presented in the study are included in the article/Supplementary material, further inquiries can be directed to the corresponding author.
