# Supplementary material for: Buffering or not working: group counseling for depression and loneliness among boarding primary school students
Source: Front Public Health. 2025 Jan 3;12:1462634. doi: 10.3389/fpubh.2024.1462634 (PMC11738940; doi:10.3389/fpubh.2024.1462634)
Supplement: Supplementary file 1 [file Supplementary_file_1.docx]

Appendix 1 Grouping counselling programme

|  | Teaching objectives | Teaching activities | Theoretical basis |
| --- | --- | --- | --- |
| Lesson 1  “*Who Am I”* | 1. Enhance the understanding of "every child is unique" in terms of four aspects: ability self, community self, physical self, and emotional self;  2. See your uniqueness, seek the support point of self-confidence, know your advantages, and learn to find foreign capital;  3. Experience the preciousness and uniqueness of life and understand the meaning of life. | 1. Meet for the first time and establish a group contract;  2. Watch the video of "Chickens Broke the Shell" and experience the preciousness of life;  3. Complete the "My Jujube" activity to experience the uniqueness of life;  4. Share the summary. | Positive Psychology: Positive Personality Traits |
| Lesson 2  *“Believe in yourself”* | 1. Learn to appreciate, accept and respect yourself, realize your advantages and enhance your self-confidence;  2. Learn to appreciate and praise others' strengths. | 1. Warm-up: Review the last class;  2. Diamond-like me: Praise myself and write out my 20 advantages;  3. High-hat bombing: Praise yourself, say and affirm each other's merits;  4. Share the summary. | 1. Positive Psychology: Positive Personality Traits;  2. Nonviolent communication |
| Lesson 3  *“Surpass*  *yourself”* | 1. Teach children how to solve and overcome difficulties;  2. Let children learn to transform ideas and turn negatives into positives. | 1. Warm-up: Review the last class;  2. My influence wheel: Let children write out the advantages and disadvantages of the people who have the greatest influence on them, and actively turn disadvantages into advantages;  3. Problem-solving equation: Give questions, brainstorm for children, choose the best solution, and present children with a general solution to the problem;  4. Share the summary. | 1. Positive Psychology: Positive Personality Traits;  2. Satir Model of Family Therapy |
| Lesson 4  *“I am the master of my emotions*” | 1. Learn to recognize emotions and identify five basic emotions;  2. Let children understand that emotions are not determined by events or others but controlled by their thoughts. Let children learn to release emotions. | 1. Warm-up: Review the last class;  2. Know five basic emotions;  3. Role-playing: Based on the ABC theory of emotions, let children understand that emotions are determined by their thoughts;  4. Paper airplane: Let children draw their emotions and release their emotions by using paper airplanes;  5. Share the summary. | Positive Psychology: Positive Emotional Experiences |
| Lesson 5  *“My future is not a dream”* | 1. Let children discover their characteristics;  2. Help children establish life coordinates, arouse children's awareness of their dreams, and cultivate their goal orientation. | 1. Warm-up: Review the last class;  2. Mould an animal: Mould an animal similar to yourself and discover your characteristics;  3. My dream board: Describe my future life, work, etc.;  4. Share the summary. | Positive Psychology: Positive Personality Traits |
| Lesson 6  *“Day Day Up”* | 1. Cultivate children's awareness of time;  2. Cultivate children's time management ability, allow children to recognize where their time is spent and to allocate limited time more effectively. | 1. Warm-up: Review the last class;  2. Crazy one minute: experience time;  3. Time pie: manage time and allocate time;  4. Share the summary. | Positive Psychology: Positive Personality Traits |
| Lesson 7  *“Long Life Road”* | 1. Guide children to experience win‒win thinking;  2. Let children experience the satisfaction and happiness brought by win‒win thinking. | 1. Warm-up: Review the last class;  2. The long road of life: role-playing as blind people, people with incomplete upper limbs, and three-legged cooperation of two people to overcome obstacles and complete the assigned tasks;  3. Share the summary. | 1. Positive Psychology: Positive Personality Traits, Positive Emotional Experiences;  2. Nonviolent communication |
| Lesson 8  *“Transposition of Listening and Speaking”* | 1. Cultivate children's ability to know themselves before knowing others;  2. Master the five levels of listening and cultivate children's empathy and effective listening ability. | 1. Warm-up: Review the last class;  2. Warm-up by art: two people keep silent and work together to complete a painting;  3. Self under the mask: draw the self outside the mask and the self inside the mask;  4. Share the summary. | 1. Positive Psychology: Positive Personality Traits, Positive Emotional Experiences;  2. Nonviolent communication;  3. Five morphological theories of Satir's model of interpersonal communication |
| Lesson 9  *“Not one less”* | 1. Emphasize the importance of the team;  2. Cultivate children's cooperation ability and understand that "unity is strength";  3. Let children realize that they are indispensable members of the team. | 1. Warm-up: Review the last class;  2. Stack pagodas: stack paper boxes skilfully as high as possible, emphasizing group cooperation;  3. Crossing the "power grid": Crossing the barrier network, the body cannot touch the "power grid", emphasizing teamwork;  4. Share the summary. | 1. Positive Psychology: Positive Personality Traits, Positive Emotional Experiences;  2. Nonviolent communication |
| Lesson 10 *“Ocean Class Power Diagram”* | 1. Help children perceive their position within the team;  2. Consciously perceive and adjust self-positioning through exploration;  3. Let children know that "all things are not mutually harmful" and help children better integrate into classes and families. | 1. Warm-up: Review the last class;  2. Watch the video "Little Rabbit of Social Fear" and share your feelings;  3. Ocean class dynamic diagram: the whole class completes the diagram on a canvas, imagines itself as a kind of creature and plant in the ocean ... and displays it on the canvas;  4. Share the summary. | 1. Positive Psychology: Positive Personality Traits, Positive Emotional Experiences;  2. Satir Model of Family Therapy |
| Lesson 11  *“It will be better tomorrow”* | 1. Cultivate children's ability to constantly update and practice;  2. Help children integrate existing resources, get ready to go, look to the future, and wish each other well. | 1. Review and sort out the contents of the first 10 lessons;  2. Organ Book: Write messages to each other and wish each other a better tomorrow. | Positive Psychology: Positive Personality Traits, Positive Emotional Experiences |

Appendix 2 Depression Scale

Instructions: Hello, classmate, this is a study on primary school students. The following question is about your personal basic information. Please tick "√" before the number that matches your situation. Please fill in your name, class and student number yourself. We will keep your information confidential. Thank you for your cooperation.

|  | Never | Sometimes | Often | Always |
| --- | --- | --- | --- | --- |
| 1. I feel pessimistic and disappointed about the future. |  |  |  |  |
| 2. I feel tired for no reason. |  |  |  |  |
| 3. I am unwilling to associate with others and have no feelings for others. |  |  |  |  |
| 4. I feel that life is boring. |  |  |  |  |
| 5. I feel like a loser. |  |  |  |  |
| 6. I hate myself. |  |  |  |  |
| 7. I feel that I am not energetic and attractive. |  |  |  |  |

Appendix 3 Loneliness Scale

|  | Never | Sometimes | Often | Always |
| --- | --- | --- | --- | --- |
| 1. It is easy for me to make new friends at school. |  |  |  |  |
| 2. I like reading. |  |  |  |  |
| 3 No one talks to me. |  |  |  |  |
| 4. I finish the task well when I am with other children. |  |  |  |  |
| 5. I often watch TV. |  |  |  |  |
| 6. It is difficult for me to make friends. |  |  |  |  |
| 7. I like school. |  |  |  |  |
| 8. I have many friends. |  |  |  |  |
| 9. I think that others cannot understand me. |  |  |  |  |
| 10. I can find friends when I need them. |  |  |  |  |
| 11. I often exercise. |  |  |  |  |
| 12. No matter what I do, it is hard for other children to like me. |  |  |  |  |
| 13. I like science. |  |  |  |  |
| 14. No one plays with me. |  |  |  |  |
| 15. I like music. |  |  |  |  |
| 16. I can get along with other children. |  |  |  |  |
| 17. I feel left out of some activities. |  |  |  |  |
| 18. I have no one to turn to when I need help. |  |  |  |  |
| 19. I like painting. |  |  |  |  |
| 20. I cannot get along with other children. |  |  |  |  |
| 21. I prefer doing things alone to working with others. |  |  |  |  |
| 22. My classmates like me very much. |  |  |  |  |
| 23. I like playing chess very much. |  |  |  |  |
| 24. I do not have any friends. |  |  |  |  |
